# Improving Classification Algorithms by Considering Score Series in Wireless Acoustic Sensor Networks

**DOI:** 10.3390/s18082465

**Published:** 2018-07-30

**Authors:** Amalia Luque, Javier Romero-Lemos, Alejandro Carrasco, Julio Barbancho

**Affiliations:** 1 Ingeniería del Diseño, Escuela Politécnica Superior, Universidad de Sevilla, 41004 Sevilla, Spain; javier@romeroyromero.es; 2 Tecnología Electrónica, Escuela Ingeniería Informática, Universidad de Sevilla, 41012 Sevilla, Spain; acarrasco@us.es (A.C.); jbarbancho@us.es (J.B.)

**Keywords:** habitat monitoring, audio monitoring, sensor network, sound classification

## Abstract

The reduction in size, power consumption and price of many sensor devices has enabled the deployment of many sensor networks that can be used to monitor and control several aspects of various habitats. More specifically, the analysis of sounds has attracted a huge interest in urban and wildlife environments where the classification of the different signals has become a major issue. Various algorithms have been described for this purpose, a number of which frame the sound and classify these frames, while others take advantage of the sequential information embedded in a sound signal. In the paper, a new algorithm is proposed that, while maintaining the frame-classification advantages, adds a new phase that considers and classifies the score series derived after frame labelling. These score series are represented using cepstral coefficients and classified using standard machine-learning classifiers. The proposed algorithm has been applied to a dataset of anuran calls and its results compared to the performance obtained in previous experiments on sensor networks. The main outcome of our research is that the consideration of score series strongly outperforms other algorithms and attains outstanding performance despite the noisy background commonly encountered in this kind of application.

## 1. Introduction

### 1.1. Sound Monitoring and Classification

Recently there has been a very significant increase in the number of devices available for monitoring and analysing environmental sounds. This increase has occurred in urban areas [1,2,3] and in environmental control operations, for example using the acoustic emission spectrum of forest fires to classify the type of forest fire [4]. The problem of analysis and classification of the sounds emitted by some species of the animal kingdom is one of the main applications of the monitoring of environmental sounds. This application has aroused great interest for experts, for several reasons.

On the other hand, the problem of classification of biological sounds can be very tricky. There are estimations that indicate that, on average, 2 min of listening are needed in 1 min of audio to be able to identify the species that emits the sound [5]. Therefore, sometimes it is not convenient to analyse manually the data provided by modern sensor networks (SN), since it is usually large volumes of data.

This is the reason why the development of intelligent systems to automate, simplify and accelerate the analysis and classification of sounds is very interesting (Ref. [6] can be consulted to find an updated review of such intelligent systems).

When approaching the process of identification of biological species, recording the different sounds in their natural habitat, using devices such as those found in [7] I the first step. It is then possible to perform a real-time processing in local mode [8] or to do so it in a remote center. This second option requires an appropriate communication system; usually a wireless sensor network (WSN), which also often includes information compression technologies [9].

The monitoring, processing and analysis of the sounds emitted by animals, used for classification, is a subject of great interest in the field of biology [10]. Particularly, the sounds emitted by the anurans are of particular interest, because they depend on climatic conditions [11]. Significant contributions have been made to the resolution of the problem of classification of anuran sounds [6,12,13].

The classification of sounds, and specifically the classification of anura sounds, is a process that has two stages. First, a set of characteristics is extracted, usually quite large. Then the classification of the sound is made, based on the extracted characteristics.

There are algorithmic techniques for extracting characteristics based on spectral or temporal parameters (for example, the spectral centroid, the bandwidth, the zero crossing rate [14,15,16], etc.). The decision to choose one or the other is made according to the application, the type of sound in question and, often, according to the preferences of the researcher. That is, these types of classification algorithms are not homogeneous, neither in the selection of characteristics or in the definition of parameters.

A concrete choice of spectral parameters is Mel Frequency Cepstral Coefficients (MFCC) [17]. They are used very extensively in the classification of sounds, and they are defined and standardized univocally [18]. This is one reason why they are a good decision to resolve the heterogeneity in the parameters.

In any case, the parameters are only the input of the techniques and classification algorithms. The problem of classifications of patterns, both sounds and other types, is a problem studied with many proposals for solutions throughout history. There are very sophisticated and efficient algorithms, capable of classifying patterns [19,20] (called automatic learning algorithms, artificial intelligence, data mining, etc.), and specifically dedicated to the sequential classification [21,22] and the classification of sound signals [23,24].

More specifically, several of the most relevant studies in bio-acoustic classification have used algorithms, such as those of k-Nearest Neighbours (k-NN), which operates on syllables modelled by entropy coefficients [25]; Decision Tree (DT), operating on sound segments (60 s in length), modelled by 14 Spectrum-Temporal Parameters (STPs) [26]; Gaussian Mixture Model (GMM), operating on sound segments (3 × 20 ms in length), modelled by Linear Frequency Cepstral Coefficients (LFCC) [27]; HMM, operating on syllables modelled by coefficients based on the Pseudo Wigner-Ville Transform (PWVT) [28]; HMM, operating on syllables modelled by MFCC [29]; HMM, operating on sound segments (made up of n 15-ms frames) modelled by MFCCs [30]; Neural Networks (NN), operating on syllables modelled by 6 STPs [31]; Neural Networks (NN), operating on syllables modelled by MFCCs [32]; Dynamic Time Warping (DTW), operating on syllables modelled by MFCC [33]; Support Vector Machine (SVM), operating on syllables modelled by 3 STPs [34]; Linear Vector Quantization (LVQ), operating on sound segments (variable length) modelled by MFCCs [35]; Singleton-type Recurrent Neural Fuzzy Networks (SRNFN), operating on frames (12 ms in length) modelled by Linear Prediction Coding (LPC) coefficients [36]; Spectral Ensemble Average Voice Print (SEAV), operating on syllables modelled by 512 Spectral Parameters (SPs) [37]; and Learning Algorithm for Multivariate Data Analysis (LAMDA), operating on syllables modelled by MFCCs [38].

In many of these studies, the sequential nature of sound is taken into consideration. Algorithms work with data that have an intrinsically sequential behaviour and are organized in syllables or segments (determined arbitrarily). One of the most important drawbacks when using sequential classification techniques is the determination of the initial and final instants of each sound.

When the algorithm is based on segments, these instants are determined by hand, using fixed length segments (which in most cases do not have a biological sense, since they do not coincide with the calls of the animals). However, if syllable-based classifiers are used, the determination of the initial and final instants of each sound is detected automatically, which is very difficult, especially if there is a lot of noise in the recording.

### 1.2. Previous Work

We have been working on the problem of classifying animal sounds for the last years, and have been working in collaboration with the Spanish Doñana National Park, where there is a sensor network used for several studies.

It is possible to perform an automatic classification of the sounds emitted by anurans, even from recordings made outdoors [39]. We have worked with 64 sound registers belonging to three different classes. The treatment with the extraction of characteristics has begun, from the 18 parameters of MPEG-7 [40]. Two simple classifiers (maximum likelihood and minimum distance classifiers) have been implemented and precise results have been obtained.

One weakness of this approach is that these good results were achieved by using ad hoc adjustment in the classifiers, which leads to the need to adapt the analysis procedure with each new data set and also the computational effort required to execute the algorithms complicates its implementation in a WSN node, which needs to work in real time.

In order to overcome these drawbacks, an alternative methodology was used in [41]. Several standard algorithms (without ad hoc adjustment) were considered in a frame classification scheme per non-sequential frame, that is, without taking into account the order of the frames, and the final labeling of a sound was achieved simply by counting the number of frames that belong to each class. It is demonstrated by experimental results that the non-sequential classification of anuran sounds is possible. The decision tree classifier showed the best performance, with a general classification success rate of 87.30%. This is a particularly good result, because the sound recordings came from a very noisy environment.

However, an attempt is made to take advantage of the information contained in the order of the frames, and six classification methods were proposed in [42], based on the machine learning domain. The database was expanded to 868 recordings from four different classes, and it was concluded that sequential classification methods can obtain a somewhat higher performance than their non-sequential counterparts. The sliding window approach with an underlying decision tree obtained the best results in the experiments, obtaining an overall accuracy of 90.48%.

On the other hand, the procedure for the extraction of anuran sound characteristics has been dealt with in [43], which compares the standardized MPEG-7, the energy of the filter bank (FBE) and the MFCC, concluding that MFCC presents the best results with an accuracy of 94.85% if the HTK version is used [44].

In [45] aspects of implementation in the environmental monitoring systems were studied, considering the time required to calculate each step in the classification process, demonstrating that it is possible to operate many anuran sound classifiers in real time, and specifically in those that they get the best classification performance.

### 1.3. Research Objectives

Although the best results in the previous works (an accuracy of about 95%) could be considered a satisfactory outcome, a more detailed analysis shows that classifiers get poor results on certain minority classes. So, new efforts have been devoted to correct this issue.

In this paper, a new algorithm is described which, while maintaining the advantages and simplicity of the non-sequential classifiers, increases its performance by employing more methods of an advanced nature than those that simply count the number of frames belonging to each class. This process will take into account not only the label assigned for the classifier to each frame, but also the scores assigned to the chance that this frame belongs to any of the classes.

The goal of the paper is to take full advantage of the information hidden in these score series in an effort to improve the classification performance.

## 2. Materials and Methods

### 2.1. WSN Architecture

It is very common to use WSNs to monitor natural habitats, in order to support biological research. The application of WSNs to the resolution of this type of problems has numerous advantages derived from the special characteristics of the sensor nodes. Their measurement capacity, data processing, computing, wireless communication and energy autonomy make it very suitable for this type of applications. On the other hand, the minimization of energy consumption and the economic cost of the nodes is a priority objective of the design, since it is desirable to have a large area of implementation and a long service life of the network.

Following these premises, a WSN has been deployed in the Doñana National Park. Its nodes have been designed considering an autonomous power supply through solar panels and low power consumption, using ARM microprocessors and low data rate transceivers. Each node incorporates an audio sensor for the identification of anuran classes and a set of meteorological sensors that measure temperature, humidity, etc., necessary to describe the climatic conditions in which the identification of the sound is made. In this work, nodes of base stations and terminal nodes have been used. There are usually very few base station nodes and many terminal nodes. The base station nodes are mainly dedicated to collecting information from the network and integrating that information into an infrastructure network (for example Ethernet, Transmission Control Protocol-Internet Protocol (TCP-IP), Long-Term Evolution (LTE) and General Packet Radio Service (GPRS).

The base station nodes act as gateways between a wireless sensor network and an infrastructure network managed by a communications service provider. That’s why they have two different network interfaces. One interface is for the infrastructure network and the other is for the wireless sensor network.

Theoretically the bandwidth of the infrastructure network could be high, but the real bandwidth is limited by the technology that is used. In this work, an architecture is proposed in which several nodes extend along a fairly large area (hundreds of km^2^), forcing us to consider the long-range radio for wireless communication.

The use of two standard bands has been chosen. The 868 MHz band will be used, (using the free radio frequency spectrum) and the 2.4 GHz band, with less vegetation penetration and shorter range, but greater bandwidth. As it is known, the data rate in these bands is few kB/s.

To address energy consumption, we consider that the nodes of the base station are next to a communication cabinet, where the connection to the infrastructure network is implemented and there is an external supply of electricity. According to this hypothesis, it is not necessary to have autonomous power generation capacity when designing the nodes of the base station.

On the other hand, it is necessary to analyze the computational capacity of the nodes, since they must be able to handle large amounts of data. The nodes execute a data fusion algorithm to minimize the size of the transmitted data, while trying to maintain the meaning of the information. That is why it is very important to be able to relate the data with the information, since they are the representation of the information. Interpreting information this way makes it possible to reduce the size of the message to be transmitted. The data measured with the sensor network comes from an audio recording, but the relevant information is the presence of an individual of specific specie in a specific audio record.

This decrease in the volume of data between the audio recording (of several kB) and the information that identifies the specific anuran detected (approximately a dozen of bytes) is very significant. To minimize energy consumption, a sound threshold is also established that activates the recognition system generating an interruption in the microprocessor that initiates a routine that addresses the acquisition and processing of the audio, so that the node only transmits information when a valid call is detected. All these decisions aim to minimize energy consumption and reduce data traffic in the wireless sensor network in communication tasks and minimize the use of the electromagnetic band, since the allowed bandwidth is few kB/s. A more detailed description of this WSN can be found in [39,43].

### 2.2. Dataset

However, for testing purposes, actual anuran sounds provided by the National Natural History Museum (Museo Nacional de Ciencias Naturales) [46] have been employed (collection code starting at FZ0496). The sounds correspond to two species, *Epidalea calamita* (natterjack toad) and *Alytes obstetricans* (common midwife toad), with a total of 868 recordings containing four classes of sounds:

1. *Epidalea calamita*; mating call (369 records).
2. *Epidalea calamita*; release call (63 records).
3. *Alytes obstetricans*; mating call (419 records).
4. *Alytes obstetricans*; distress call (17 records).

A total of 4343 s (1 h 13 min) of recordings have been analysed, with an average duration of 5 s. The sounds have been recorded in five different locations (four in Spain and one in Portugal) using a Sennheiser ME80 microphone. They are subsequently sampled at 44.1 kHz.

**Figure 1:**
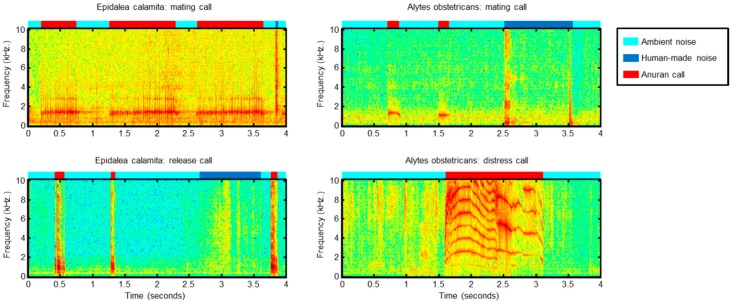
Spectrograms of sample calls for each sound class.

A common feature of all the recordings is that they have been taken in their natural habitat, with very significant surrounding noise (wind, water, rain, traffic, voices, etc.), which posed an additional challenge in the classification process. Building noise-robust recognition systems is a topic thoroughly addressed in the literature for human speech [47,48], bioacoustics monitoring [49], and anuran calls [50]. Figure 1 depicts the spectrograms of a sample call for each class indicating the segments containing ambient or human-made noises and anuran calls.

In order to guarantee the validity of the results, a cross-validation technique is applied. First the dataset is randomly sorted and then split into k-folds. Several of these folds are used to obtain the parameters of the classifiers (training dataset), whilst others (validation dataset) are employed to determine the hyper-parameters (if any) of the models. The remaining folds (testing dataset) are used to test the classifiers and to estimate their performance. In this paper, the original dataset has been divided into seven folds, using 5-folds for training (approximately 70% of the sounds), one fold for validation (15%), and one fold for testing (15%).

**Figure 2:**
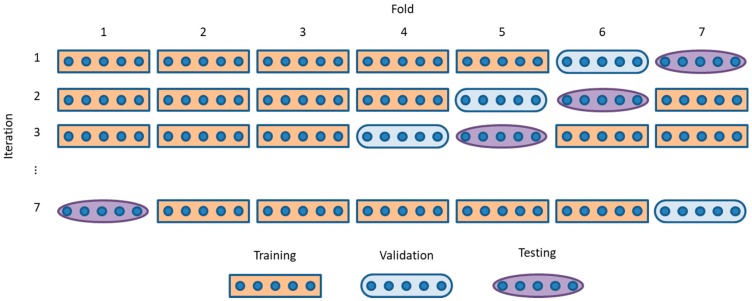
Cross-validation technique.

However, the cross-validation technique also implies cyclically shifting the folds dedicated to every purpose so that, after k iterations, every element is used five times for training, once for validation, and once for testing. The overall performance is estimated as the mean performance obtained in every iteration. The overall process is depicted in Figure 2.

For each of the sound records used in the training phase, not only does its class have to be identified, but also the regions-of-interest (ROIs) in the sound, that is, the frames that really belong to that class and are neither silence nor noise. In order to determine the ROIs, the experts listen to the recordings of the anuran calls and simultaneously consider the spectrogram, and label each frame that they consider may belong to any of the possible classes. In the cross-validation technique, every record will be used for training in some iteration, so that the ROIs for the whole 868 recordings have been determined. Table 1 summarizes the dataset of the sounds.

### 2.3. Feature Extraction

The first step in the classification of a sound is to represent it using some kind of mathematical description. Most of these descriptions are directly or indirectly based on the spectrum of the signal and its evolution over time, which is usually known as the spectrogram. Certain approaches to sound classifications make a straight use of the spectrogram in the so-called featureless classification. Although this is a simpler and straightforward approach, it usually requires more computational resources and, therefore, renders it unsuitable for implementation in the low-cost and low-power nodes usually employed in sensor networks.

For this reason, the most common approach is to extract a bundle of features which represent the sound using many fewer values, thereby permitting a considerably faster classification. This process starts by splitting the sound up into frames of fixed duration. In the case of vocal sounds, this duration is usually related to the mechanism of the production of the sound and, specifically, to the period of the opening and closing of the vocal cords, which is approximately 10 ms, both in humans [51] and in anurans [52]. The framing process always introduces a distortion in the sound spectrum. In order to decrease this undesired effect, it is common to use a wider window (25 ms in this case), to move the window forward in a shorter hop size (10 ms in this paper), and also use a bell-shaped window function (the Hamming window for our research). In this approach, each frame is defined by 1102 values (25 ms sampled at 44.1 kHz) and it overlaps with the sides of the adjacent frames.

Once each frame has been obtained, it is represented using a few parameters (features). In previous work, two alternatives have been considered and compared: the standard MPEG-7 parameters and those of the MFCC. We have concluded that MFCC alternative clearly obtains better classification performances [43] and is more computationally efficient [39]. This is therefore the approach used in this research and, more specifically, the solution provided in the Hidden Markov Model Toolkit (HTK) [44], a widespread implementation originally developed by Cambridge University. The MFCC feature extraction process, using the HTK by-default options, can be outlined in the following steps:

1. Sound pre-emphasis, using a first-order digital filter with constant α=0.97, which provides a more uniform signal-to-noise ratio (SNR).
2. Sound framing using a Hamming window of 25 ms and a hop size of 10 ms.
3. Obtaining the Energy Spectral Density (ESD) of each frame.
4. Representing the values of ESD in logarithmic scale (LogESD).
5. Spectrum filtering between 300 Hz and 3700 Hz, a band where the vocal sounds contain most of their energy.
6. Obtaining the Mel Logarithmic Filter Bank Energy (MelLogFBE) spectrum as the LogESD at a triangular filter bank which uses 20 filters centred at the Mel frequencies [53]. After this step, the frame spectrum is represented by the 20 values of the energy at each filter.
7. Cepstral representation of the MelLogFBE using the Discrete Cosine Transform, obtaining 20 cepstral coefficients (MelLogDCT), which are the first form of the MFCC.
8. Reducing the number of cepstral coefficients (MFCC) by preserving the D=13 first coefficients and discarding the remaining 7.
9. Cepstral liftering the MFCC using a sine lifter (a filter in the cepstral domain) with constant L=22.

A more detailed description of the feature extraction process can be found in [43].

### 2.4. Frame Classification

Once each frame has been represented using the MFCC features, it can then be classified as belonging to any of the four classes (anuran calls) under study or to none of them (silence/noise). To this end, eight supervised classifiers have been employed: minimum distance (MinDis) [54], maximum likelihood (MaxLik) [55], decision trees (DecTr) [56], k-nearest neighbours (kNN) [57], logistic regression (LogReg) [58], neural networks (Neur) [59], discriminant function (Discr) [60], and Bayesian classifiers (Bayes) [61].

Prior to classification, the values of the 13 features are normalized. In this respect, the mean $\mu_{j}$ and the standard deviation $\sigma_{j}$ are obtained for the j-th feature on considering all the frames in the training dataset. The value $x_{ij}$ of the j-th feature at the i-th frame is then normalized as:

(1) $$\;x_{ij}^{\prime }=\frac{x_{ij}-\mu_{j}}{\sigma_{j}}.\;$$

Although the concluding results have to be implemented in the SN nodes, a previous desktop prototype has been designed to perform the comparisons in the classification algorithms. For this reason, the eight aforementioned classifiers have been prototyped using MATLAB (2014a, Mathworks, Natick, MA, USA). The minimum-distance classifier in its training phase obtains the mean value $\mu_{jk}^{\prime }$ for the j-th normalized feature belonging to the k-th class. In the test phase for every frame, the distance $d_{k}$ between the frame features and the mean value of the k-th class is obtained in accordance with the expression:

(2) $$d_{k}=\sqrt{\sum_{j=1}^{D}{\left(x_{j}^{\prime }-\mu_{jk}^{\prime }\right)}^{2}},$$

where $x_{j}^{\prime }$ is the value of the j-th normalized feature. The class assigned to the frame is that with the minimum distance.

The maximum likelihood classifier is used under a mix of two Gaussian probability distributions with full covariance. The neural network classifier is based on a feed-forward neural network with a 10-neuron hidden layer and a 1-neuron output layer. The remaining methods and classifiers have been coded based on built-in MATLAB functions using their default parameters, which are reflected in Table 2. A more detailed description of the classifiers employed can be found in [40].

From the previously described procedure, each frame of a sound is classified in one of the C+1 possible classes (C anuran calls, plus the silence/noise class). The final labelling of the recording can then be decided by simply counting the number of frames belonging to each class (not considering the silence/noise frames).

### 2.5. Score-Series Classification

**Figure 3:**
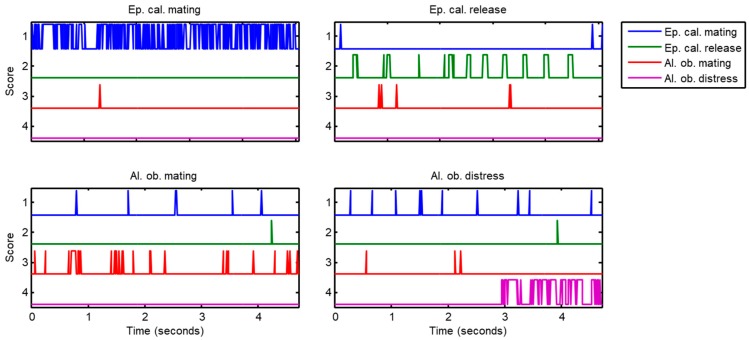
Score series of an example call for each sound class.

The aforementioned counting technique is probably the most straightforward approach for the classification of a sound, considering that their frames have previously been classified. However, a more insightful method is possible by considering that frame classifiers can offer not only a label deciding the class of the frame, but also information of a more precise nature that assigns a score $s_{ik}$ to the feasibility that the i-th frame belongs to the k-th class. This score is usually (but not always) the probability that the i-th frame belongs to the k-th class. Although C+1 score values (one for each class) are obtained in each frame, only C of them are relevant, because the last value can be obtained as a function of the other values. If the score values represent probabilities, they have to add up to 1. Figure 3 depicts the scores over the time $s_{k}\left(t\right)$ of an example call for each class (C=4) when they are classified using a kNN algorithm.

**Figure 4:**
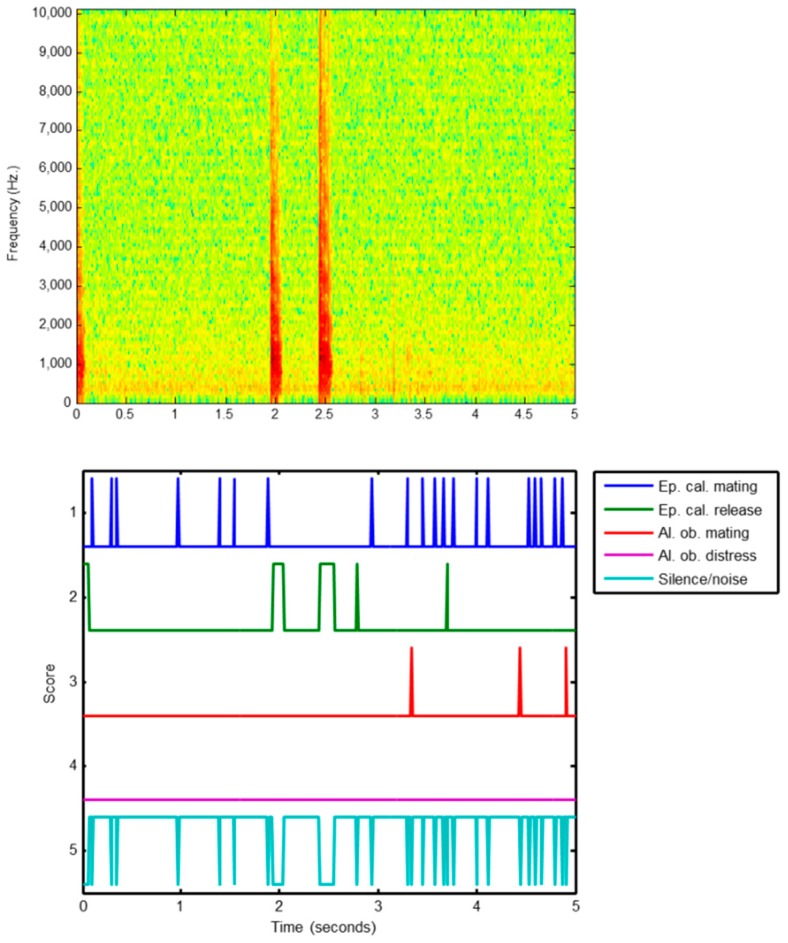
Example of misclassification.

The frame classification of a sound therefore produces C score series $s_{k}\left(t\right)$ or, equivalently, a C-dimension score vector series S(t). Intuitively, score series should carry more information than simply the frame label. This additional information could therefore be used to improve the classification process by substituting the frame label count by a more thorough score-series classification. Let us consider the example of a misclassified *Epidalea calamita* release call as represented in Figure 4. 

The upper part of that figure shows the spectrogram of three release calls, centred at 0, 2 and 2.5 s, respectively. In the lower plot, the score series are depicted by applying a kNN classifier to every frame. It can be seen that the frames corresponding to the calls are correctly identified (score series 2 in green). Additionally, most of the noise/silence frames are correctly classified (score series 5 in cyan), but a small proportion of them are misclassified as mating calls (score series 1 in blue). Since the duration of the release calls is very short, the number of frames correctly labelled as release calls is lower than the number of noisy frames misclassified as mating calls. Finally, the sound is (incorrectly) classified by counting the number of frames belonging to each class.

In that figure, it is clear that a deeper insight on score series is possible than just counting frame labels. To this end, the first step should be how to represent the score series. For this purpose, we have adapted the MFCC features to the special case of the score series.

Firstly, while the sound values are initially windowed in 25 ms frames, the score series are considered as a whole because they have a similar number of values. Indeed, a 25 ms frame of a sound sampled at 44.1 kHz contains 1102 values while, on the other hand, a 5 to 10 s sound contains 500 to 1000 frames (considering a 10 ms hop size): a figure in the same order of magnitude.

Moreover, since the score series are definitely not sounds, then neither the SNR flattening pre-emphasis nor the spectrum filtering nor the Mel scaling nor the cepstral liftering have any physical sense. The process of feature extraction from the score series can therefore be described as the following steps:

1. Obtaining the Energy Spectral Density (ESD) of the score series.
2. Representing the values of ESD in logarithmic scale (LogESD).
3. Obtaining the Linear Logarithmic Filter Bank Energy (LinLogFBE) spectrum as the LogESD at a triangular filter bank which uses 20 filters centred at linear (not Mel scaled) frequencies. After this step, the frame spectrum is represented by the 20 values of the energy at each filter.
4. Cepstral representation of the LinLogFBE using the Discrete Cosine Transform, obtaining 20 cepstral coefficients (LinLogDCT), which are the first form of the Linear Frequency Cepstral Coefficients (LFCC).
5. Reducing the number of the cepstral coefficients (LFCC), by preserving the D=13 first coefficients and discarding the remaining 7.

**Figure 5:**
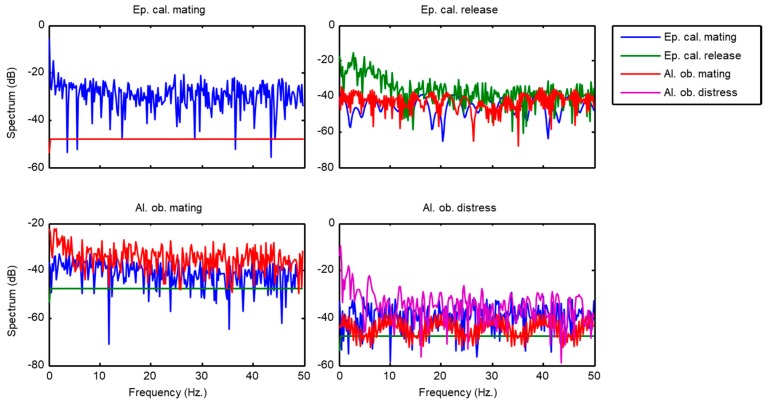
Energy Spectral Density corresponding to score series of an example call for each sound class.

Figure 5 depicts the LogESD of the score series of an example call for each class (C=4) when they are classified using a kNN algorithm. Each ESD representation has up to 4 spectrums (one for each class). In several examples, some of the spectrums are not shown, and these correspond to cases when the score is zero in any frame, thereby resulting in a null energy at every frequency, which gives a minus infinity value in the logarithmic scale.

**Figure 6:**
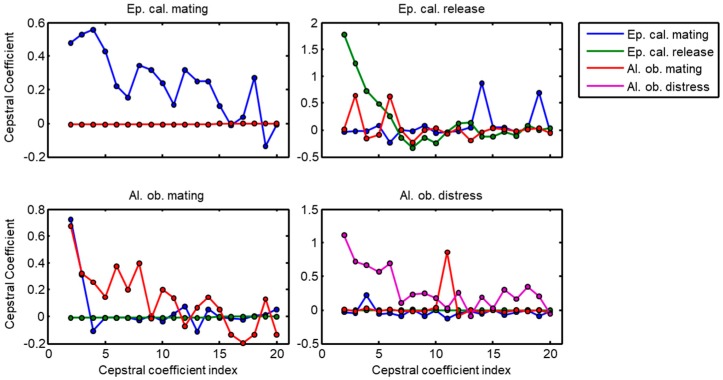
Linear Frequency Cepstral Coefficients (LFCC) corresponding to score series of an example call for each sound class.

Figure 6 depicts the LFCC of the score series of a sample call for each class (C=4) when they are classified using a kNN algorithm. Again, each LFCC representation has up to four spectra (one for each class). In several examples, a number of the LFCC series are not shown, which correspond to cases when the LogESD has the minus infinity value.

**Figure 7:**
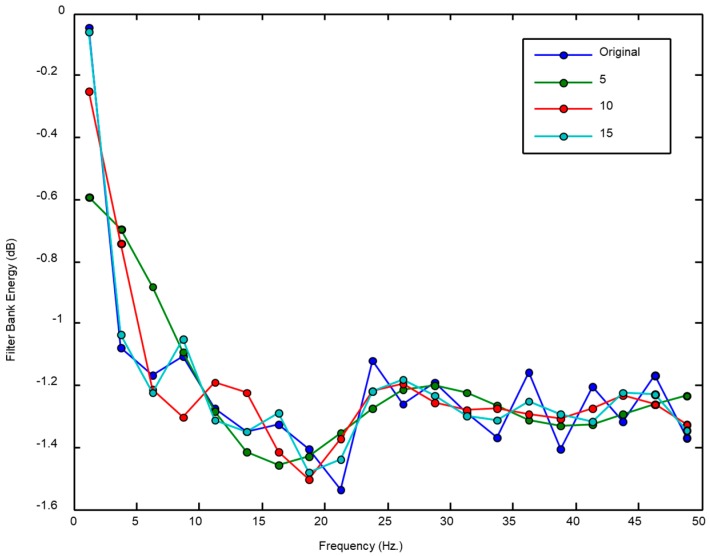
Impact on the spectrum of reducing the number of the cepstral coefficients.

Reducing the number of the cepstral coefficients certainly exerts a certain impact on the accuracy of the LinLogFBE representation. As an illustration, Figure 7 compares the original LinLogFBE spectrum (using 20 coefficients) to those obtained using a lower number of cepstral coefficients.

**Figure 8:**
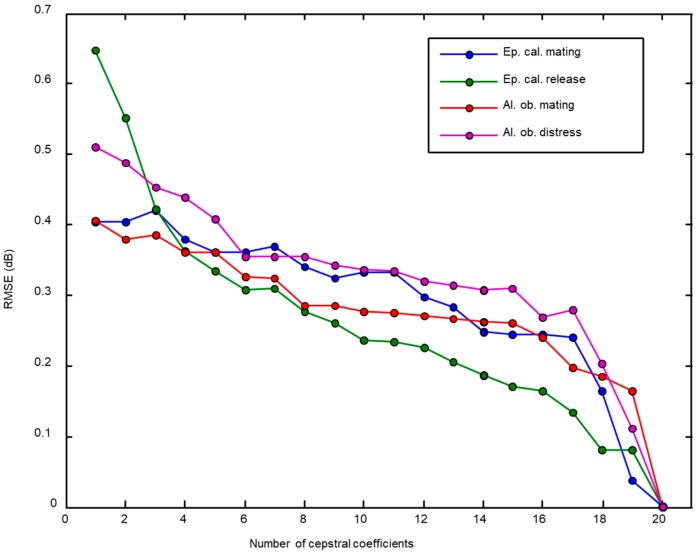
RMSE of representing the score series vs. the number of cepstral coefficients for an example call of each sound class.

The impact of reducing the number of cepstral coefficients can also be analysed by measuring the Root Mean Square Error (RMSE) which represents the LinLogFBE spectrum with a different number of cepstral coefficients (LFCC). The results for several examples of different classes are depicted in Figure 8.

**Figure 9:**
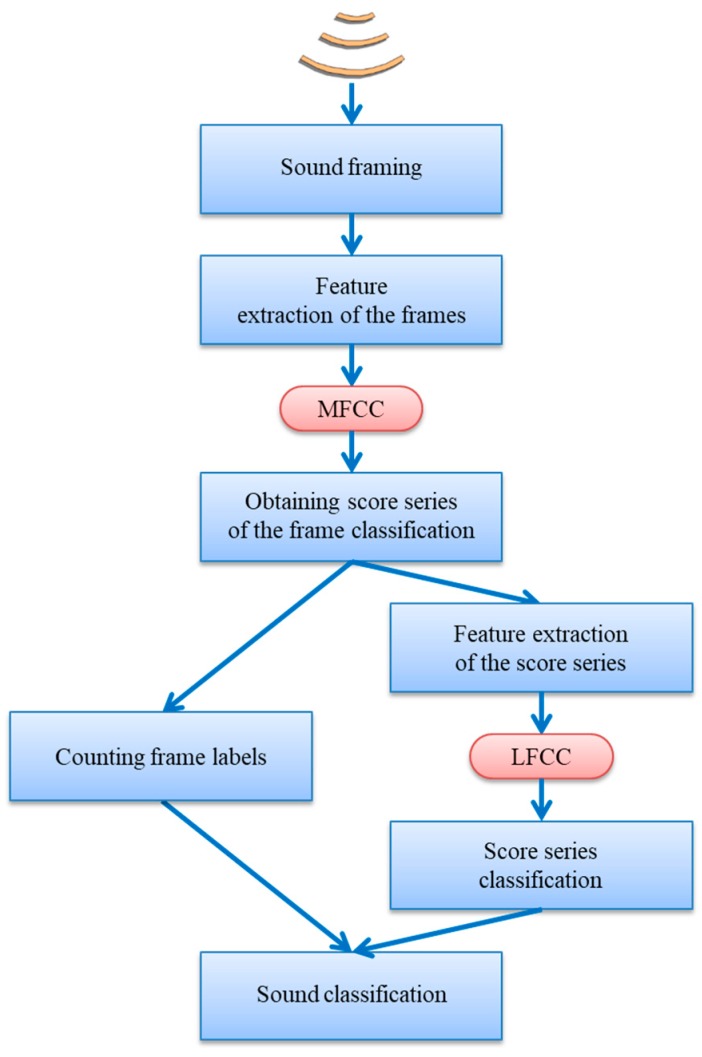
General schema for the classification procedure.

The overall process of representing score series yields a set of 13 LFCC features for each class. In our case therefore, the score series of every sound are represented using 52 (13 × 4) features. These features can now be used to classify the sound using the cross-validation technique and the same classifiers as described in Section 2.3. The general schema of the proposed procedure is depicted in Figure 9.

### 2.6. Classification Metrics

One important issue that has to be addressed in the process of designing classification algorithms involves how to measure their performance. One of the most widely used methods to perform this task is through the confusion matrix defined as:

(3) $$\;CM\equiv \left[\begin{array}{cccc} m_{11} & m_{12} & \ldots & m_{1C}\\ m_{21} & m_{22} & \ldots & m_{2C}\\ \vdots & \vdots & \ddots & \vdots \\ m_{C1} & m_{C2} & \ldots & m_{CC} \end{array}\right],\;$$

where $m_{ij}$ represents the number of elements of the i-th class labelled by the classification algorithm as belonging to the j-th class, and C is the total number of classes.

**Figure 10:**
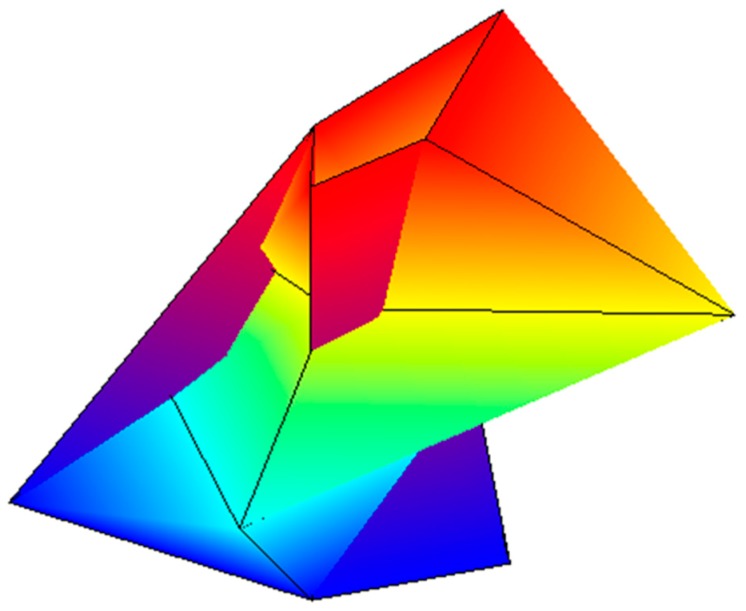
Artistic representation of a multiclass confusion matrix.

Classification performance can be seen as a polyhedral entity which is not easy to reduce to a single measure, as shown in Figure 10, which depicts an artistic representation of a multiclass confusion matrix. There is no single way to select the best algorithm as any of them can obtain good results in one class but poor scores in other classes.

For this reason, several metrics are usually considered which permits the polyhedral characteristics of the classification performance to be viewed from different points of views. The most relevant metrics and their definitions are shown in Table 3, where they are first computed for each class and then an average value is obtained as a global value for the algorithm classification performance [62,63]. In the table, the term $m_{i}$ represents the total number of elements actually belonging to the i-th class, while $e_{i}$ stands for the number of elements labelled by the classification algorithm as belonging to the i-th class.

All these metrics take values in the [0,1] range, except the last three whose ranges lie in the [−1,1] interval. For comparison purposes, these metrics will be used in their normalized version. By naming a metric defined in the [−1,1] interval as μ, it can be normalized in the [0,1] range by the expression:

(4) $$\;\mu_{n}\equiv \frac{\mu +1}{2}.\;$$

Although the full set of metrics defined in Table 3 will be considered in this paper, the $F_{1}$ score will be used whenever a single metric has to be selected. This selection is mainly due to the fact that $F_{1}$ score combines two perspectives (sensitivity and precision) in a single metric, which makes it one of the most widely used metrics in the literature.

### 2.7. Bootstrap Analysis

Once the classification performance metrics are obtained, it is good practice to estimate the confidence interval of their values. To undertake this task, a bootstrap analysis is performed [64], by firstly considering the testing dataset T that contains S sounds. From this dataset, S samples are then taken with replacement and a new $T_{1}$ dataset is obtained. Due to the replacement in the sampling process, certain sounds are not contained in $T_{1}$, while others are repeated at least once. The classification metric vector $\mu_{1}$ can now be computed for the $T_{1}$ dataset.

This process is repeated $N_{b}$ times (usually a large number), thereby obtaining datasets $T_{1},T_{2}\cdots T_{N_{b}}$ and their corresponding metric vectors $\mu_{1},\mu_{2},\cdots \mu_{N_{b}}$. This set of metric vectors is employed to estimate the probability density function (pdf) of the metric vector f(μ) and other related statistics. This procedure is commonly employed to derive the confidence interval of the classification metrics. Therefore, by considering the metric $\mu_{k}$, which is the k-th metric in the μ vector, and its pdf $f_{k}\left(\mu_{k}\right)$, the confidence interval of $\mu_{k}$, for a given confidence level γ, is the interval between the values $u_{k}$ and $v_{k}$ such that $\mathrm{Pr}\left[u_{k}\leq \mu_{k}\leq v_{k}\right]=\gamma$. The value of $u_{k}$ can be estimated as the γ/2 percentile of $\mu_{k}$, and the value $v_{k}$ as the 100−(γ/2) percentile. Throughout this paper, bootstrap analysis with $N_{b}=10,000$ and a confidence level of γ=95% is used.

Bootstrap analysis can also be employed to estimate the probability that a certain metric outperforms another. For every $T_{j}$ dataset, the classification methods 1 and 2 are employed and their metric vectors $\mu_{j1}$ and $\mu_{j2}$ are computed. The difference between these metric vectors is then derived by $\delta_{j}=\mu_{j1}-\mu_{j2}$. The probability density function (pdf) of the vector of differences f(δ) and the continuous density function (cdf), F(δ), can then be computed. Finally, by considering the difference $\delta_{k}$, which is the k-th metric in the δ vector, and its cdf $F_{k}\left(\delta_{k}\right)$, the probability of outperforming, $o_{k}$, is the probability that $\delta_{k}>0$, that is, $o_{k}=\mathrm{Pr}\left[\delta_{k}>0\right]=\;F_{k}\left(0\right)$.

## 3. Results

### 3.1. Classification by Counting Frames

**Figure 11:**
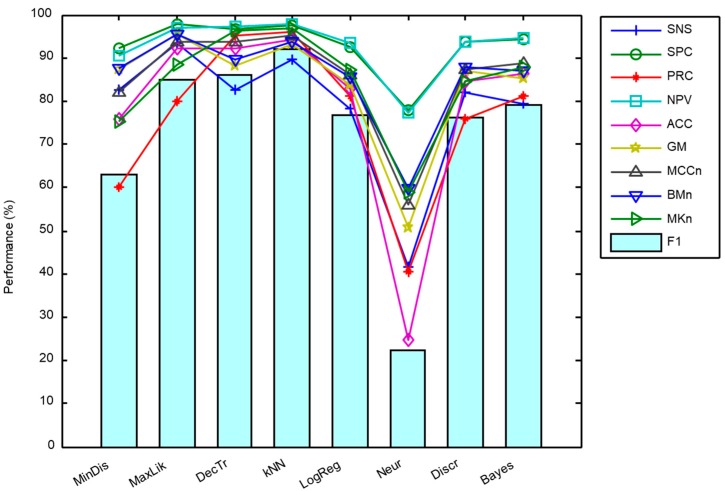
Performance metrics for the procedure of counting frames.

The sound classification procedure described in Section 2.3 has been applied to the dataset presented in Section 2.1 once its MFCC features had been extracted according to Section 2.2. The results have been measured using the metrics described in Section 2.5 and are presented in Figure 11 and in Table 4.

**Figure 12:**
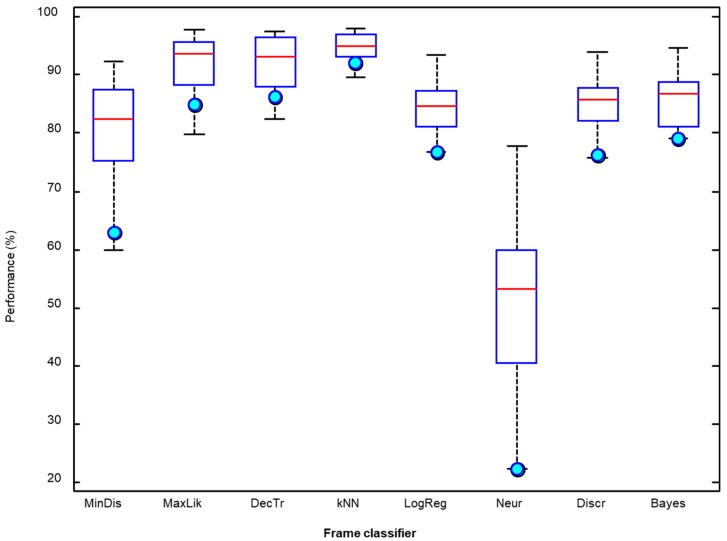
Boxplot of the performance metrics for the procedure of counting frames.

It can be seen that the kNN classifier offers the best results for six out of the 10 metrics considered (with the F1 score featuring among these metrics), while the maximum likelihood algorithm outperforms the other algorithms for the remaining four metrics. Additionally, kNN requires much less in computing resources than the maximum likelihood algorithm classifier [45]. Moreover, the values of the kNN metrics have less spread as can be observed in the boxplot depicted in Figure 12 where the light blue circle indicates the F1 score. 

For these reasons, the kNN algorithm will be considered the best classifier for the procedure of counting frames, since it obtains a remarkable 94% accuracy despite the noisy background of many recordings. A more detailed consideration of the classification results (see Table 5) reveals that, although the overall results are good, they present poor performance in classifying the *Epidalea calamita* release call, whereby more than one third of the calls are misclassified, mainly as *Epidalea calamita* mating calls (30%). An example of this misclassification was presented in Figure 4.

### 3.2. Classification of Score Series Obtained with the kNN Frame Classifier

**Figure 13:**
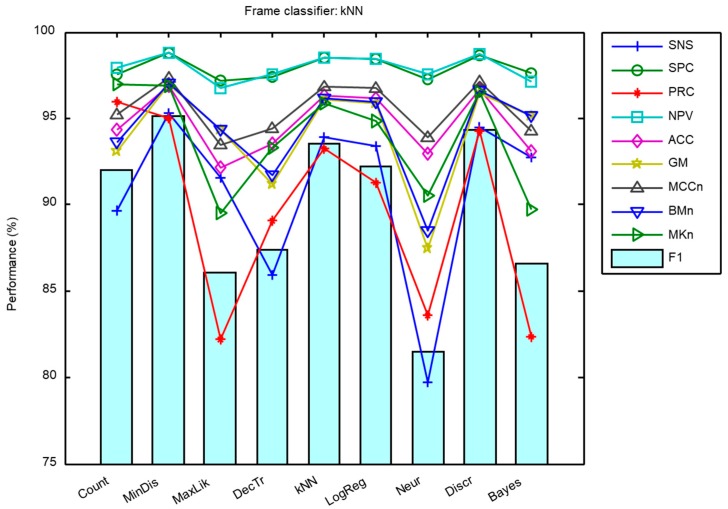
Performance metrics classifying the score series obtained by the kNN frame classifier.

As described in Section 2.4, labelling sounds by just counting frame labels is not intuitively the best procedure. As an alternative therefore, we have explored the classification of the score series obtained by the kNN algorithm selected in the previous section. After extracting the LFCC features of the score series, they are processed using the same eight classification algorithms and the results obtained are depicted in Figure 13 and in Table 6, where the counting method is also shown for comparison purposes.

**Figure 14:**
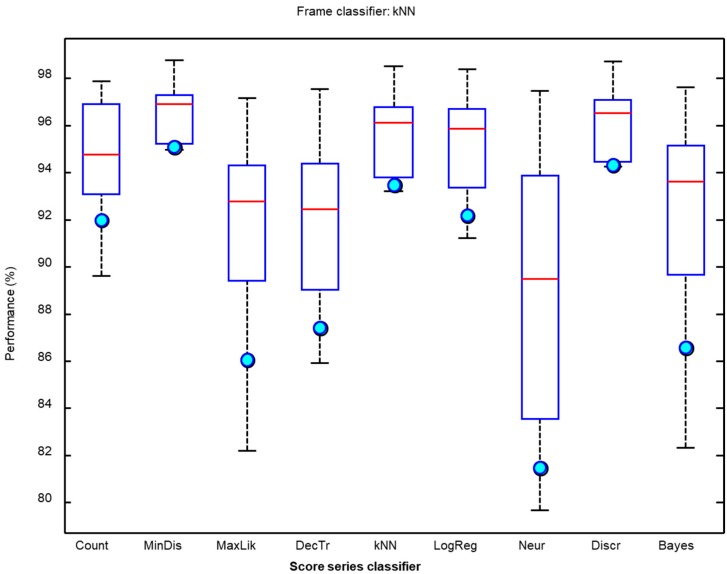
Boxplot of the performance metrics for the different classifiers of score series operating on the results obtained by the kNN frame classifier.

It can be seen that the minimum-distance classifier operating on the score series obtained by the kNN frame classifier offers the best results for 9 out of the 10 metrics considered (with the F1 score among them). Additionally, the minimum-distance classifier provides a very convenient classifier in terms of the computing resources required [46]. Moreover, the values of the metrics of the minimum-distance score classifier have less spread than other classifiers of score series as can be seen in the boxplot depicted in Figure 14 where the light blue circles indicate the F1 score. 

### 3.3. Optimum Classification of Score Series 

In the previous section, the frame classifier and the score-series algorithm were separately optimized, that is, firstly the frame classifier was determined and the optimum score-series algorithm was subsequently derived. 

**Figure 15:**
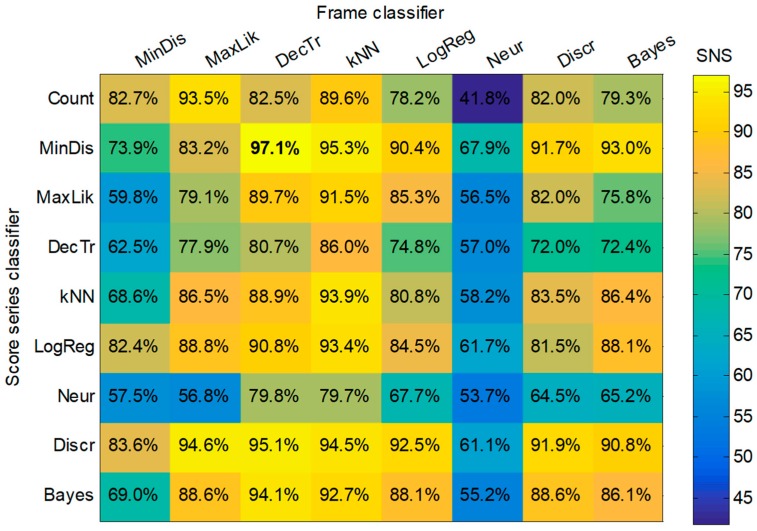
Matrix of SNS values for every pair (score, frame) classifiers.

However, it is also possible to run a joint optimization process to simultaneously seek the optimum values for both methods. By running this process, a matrix for each performance metric is obtained showing a value for every pair defining the (score, frame) classifiers. The matrix for the first of the metrics (SNS) is depicted in Figure 15. Similar matrices can be obtained for the remaining performance metrics.

**Figure 16:**
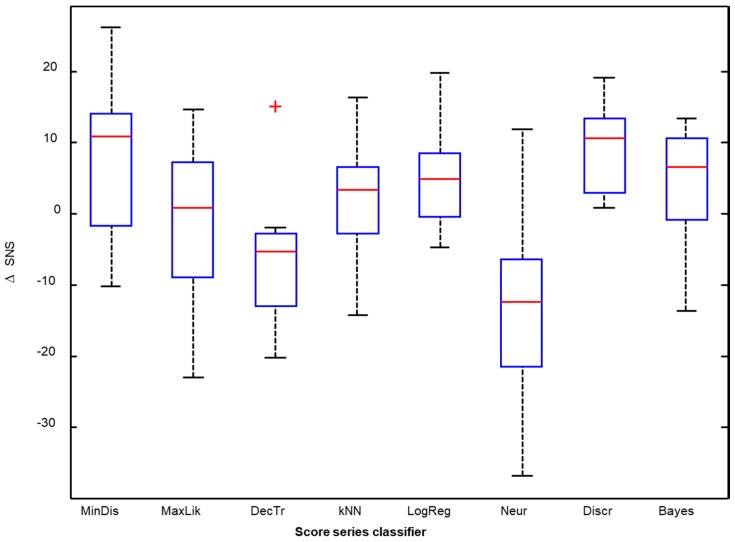
Improvement of the SNS obtained for each score-series classifier.

Applying a score-series classifier usually (but not always) outperforms the method of counting frames. The improvement of the SNS metric by applying, for instance, the minimum-distance score classifier to every frame classifier constitutes the difference between the second and the first row of the matrix in Figure 15. These values can be drawn in a boxplot as shown in the first box of Figure 16. The improvement of the SNS obtained for the remaining score-series classifiers are represented in the remaining boxes in the figure.

It can be seen that the minimum distance and also the discriminant function enhance the SNS by approximately 10 points (median value) compared to the counting frame method. Similar results can be obtained for the remaining performance metrics.

**Figure 17:**
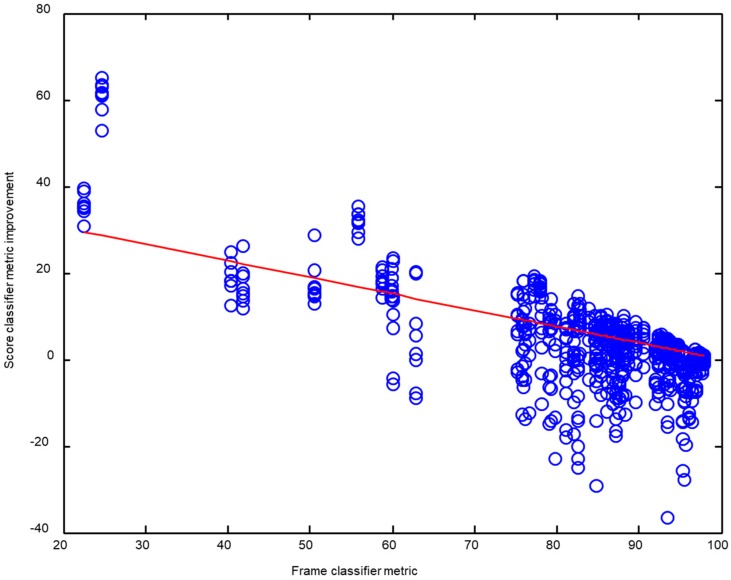
Improvement of the performance metric obtained by the score-series classifier vs. the original (frame classifier) metric.

As should be expected, the improvement in performance metrics obtained by the score classifiers is greater when the original metric (obtained from the frame-counting procedure) has a lower value. In other words, it is easier to enhance poor results than good results. To show this effect, the improvement of every performance metric (10 values) for each pair (score, frame) classifier (8 × 8 values) is depicted in Figure 17 vs. the original metric (obtained counting frames after a frame classification). A total of 640 values have been obtained and its regression line (with a slope of −0.376) is also shown.

**Figure 18:**
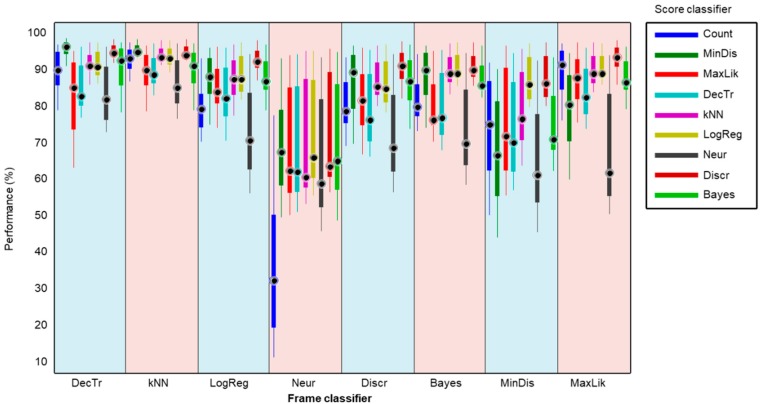
Boxplot of the performance metrics for the different score-series classifiers operating on the results obtained by every frame classifier.

In order to obtain an in-depth insight into the performance of each pair of (score, frame) classifiers, all the metrics (not just the SNS as in Figure 15) should be considered. The direct application of this approach would produce a cube with a 3D matrix of values for every triad (score, frame, metric). This cube is difficult to represent and interpret and therefore a different plotting method should be pursued. The alternative method used is to depict a boxplot representing the metric values of each pair of (score, frame) classifiers, but drawn one-dimensionally. The result is shown in Figure 18, where, first the decision-tree frame classifier is considered (light blue band to the left), followed by the application of each of the nine score classifiers (including the frame-counting method); then, for each score classifier, the ten metrics are employed to build a boxplot. Subsequently, every frame classifier is considered. For each pair (score, frame) of classifiers, four elements representing its metrics are drawn: a filled box from the 25% to 75% percentiles of the values of the metrics; an upper vertical line from the 75% percentile to the maximum; a lower vertical line from the 25% percentile to the minimum; and a black filled circle corresponding to the median value.

**Figure 19:**
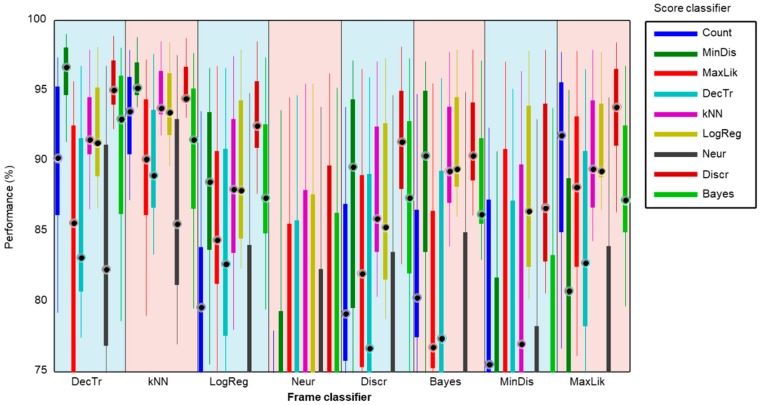
Boxplot (detail) of the performance metrics for the different score-series classifiers operating on the results obtained by every frame classifier.

For a better comparison, a detail of this graph is depicted in Figure 19. There it can be observed that the best results are achieved by a minimum-distance classifier (dark green box) operating on the score series obtained with a decision-tree frame classifier (light blue band on the left).

**Figure 20:**
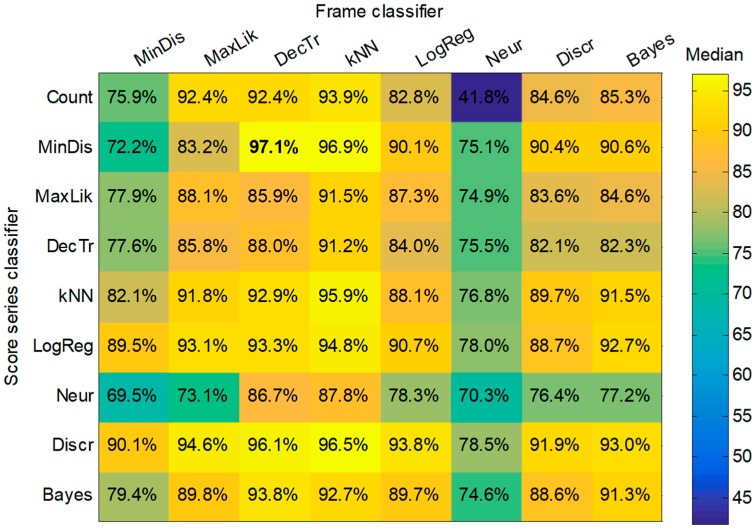
Matrix of the median value of performance metrics for every pair (score, frame) classifier.

By considering only the median value of the performance metrics, a matrix of values can be built for every (score, frame) classifier. The result is shown in Figure 20, and verifies the (minimum distance, decision tree) as the best pair of classifiers.

The confusion matrix obtained using this pair of classifiers is shown in Table 7, which reveals that the misclassification problem of the *Epidalea calamita* release call has been solved, while the good results for the remaining sound classes remain largely unaltered, with an outstanding accuracy of 97.35%.

### 3.4. Bootstrap Analysis

In the previous subsections, several classification methods have been identified. Firstly, the kNN is the best frame classifier when the counting method is used. Later, when considering score-series classifiers, the decision-tree frame classifier has shown itself to be the most efficient. These two classifiers have been used as the baselines for the determination of the improvement achieved using other procedures. Finally, the joint optimization of the frame classifier and the score-series classifier leads to the detection of an optimum method: the (minimum distance, decision tree) as the best pair of classifiers. Table 8 summarizes the performance metrics of these three classification methods.

**Figure 21:**
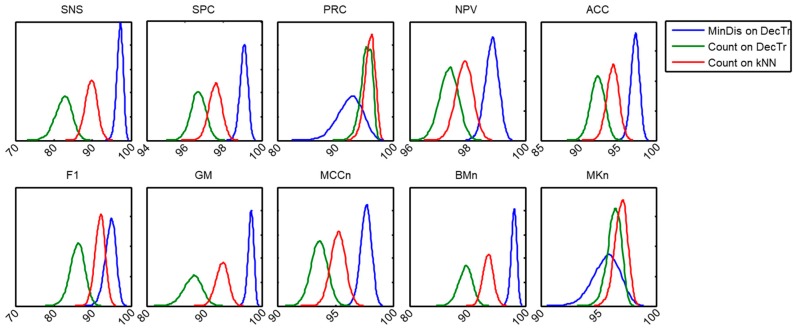
Probability density function of each performance metric for 3 selected pairs of (score, frame) classifiers.

Using bootstrap analysis, the probability density function of each performance metric for each pair of (score, frame) classifiers can be estimated. The results regarding the ten metrics for the three previously selected pair of classifiers are shown in Figure 21. It can be seen that for eight out of 10 metrics, the (MinDis, DecTr) pair obtains the best results and, additionally, its outperformance is robust (the pdf graphics barely overlap).

By means of considering not only the mean value of the improvements but also their statistical distribution, the confidence interval for each metric and method can be derived. These results are shown in Table 9, where the probabilities that the (MinDis, DecTr) pair outperforms the simpler methods (Count, kNN) and (Count, DecTr) pairs are also presented. It can be seen that for almost every metric, the selected method obtains a clear performance improvement with a high probability, that is, the outperformance is robust.

## 4. Discussion

The preceding results first show that classifying score series clearly and robustly outperforms the method of simply counting labels obtained after the frame classification phase. Good results can be obtained with several score-series classifiers, and outstanding results are attained from the minimum-distance and the discriminant-function algorithms.

As it should be expected, the improvement in performance metrics obtained by the score classifiers is greater when the original metric (obtained through the procedure of frame-counting) has a lower value. In other words, it is easier to enhance poor results than good results. This dependence is approximately lineal, with an improvement of about 4 points for performance metrics of 90% and, therefore, eight points of improvement when the metric value is 80%.

Through this analysis, it has been shown that score series can successfully be represented by their Linear Frequency Cepstral Coefficients (LFCC), an adaptation of the MFCC (features originally designed to represent human vocal sounds) to the special case of the score series which are definitely not sounds.

It has been found that the optimum classifier (MinDis, DecTr) increases the F1 score by approximately 9 points and obtains a noteworthy overall accuracy of 97.35%. Since the level of background noise in the recordings is high, this can be considered a remarkable result. Moreover, the confusion matrix for this method shows that the good performance is fairly balanced among classes.

Furthermore, from the results, the decision-tree method (and also kNN) appears as one of the best frame classifiers. This fact is consistent with other studies where non-speech sounds [65], or more specifically, environmental sounds [66] are considered.

The outperformance using these methods may only be moderate (mainly for the best frame classifiers) but it is reliably consistent. The probability that the selected score-series classifier improves its counting-frame-label counterparts is extremely high (more than 98% in most cases). 

On the other hand, the cost of computing of a more complex nature due to the double classification process has been considered in detail [45]. The real-time processes required in the second (score-series) classification process involve extracting the LFCC features of the score series and then classifying said features. The extraction process of a 5-s score series takes approximately 40 microseconds per class measured on a conventional desktop computer, that is, about 150 microseconds in our 4-class research. Moreover, the time required for the minimum-distance classifier to label a 52-feature vector representing the 5-s sound segment is of about 50 microseconds, while the discriminant function requires only half of this time. These times (200 microseconds) are negligible compared to the sound length (5 s, 25,000 times higher).

## Figures and Tables

**Table 1:** Dataset of sounds.

Sound Class	Sound Recordings		
	Number	Seconds	Ratio
*Ep. cal.* mating call	369	1853	43%
*Ep. cal.* release call	63	311	7%
*Al. ob.* mating call	419	2096	48%
*Al. ob.* distress call	17	83	2%
Total	868	4343	100%

**Table 2:** MATLAB functions supporting the various classifiers.

Classifier	Training Functions	Test Functions
MinDis	-	-
MaxLik	fitgmdist	mvnpdf
DecTr	fitctree	predict
kNN	fitcknn	predict
LogReg	mnrfit	mnrval
Neur	feedforwardnet; train	net
Discr	fitcdiscr	predict
Bayes	fitNaiveBayes	posterior

**Table 3:** Classification performance metrics.

Metric	i-th Class	Global
Sensitivity SNS	$\frac{m_{ii}}{m_{i}}$	$\frac{\sum_{i=1}^{C}SNS_{i}}{C}$
Specificity SPC	$\frac{\sum_{\begin{array}{c} u=1\\ u\neq i \end{array}}^{C}\sum_{\begin{array}{c} v=1\\ v\neq i \end{array}}^{C}m_{uv}}{\sum_{\begin{array}{c} u=1\\ u\neq i \end{array}}^{C}m_{u}}$	$\frac{\sum_{i=1}^{C}SPC_{i}}{C}$
Precision PRC	$\frac{m_{ii}}{e_{i}}$	$\frac{\sum_{i=1}^{C}PRC_{i}}{C}$
Negative Predictive Value (NPV)	$\frac{\sum_{\begin{array}{c} u=1\\ u\neq i \end{array}}^{C}\sum_{\begin{array}{c} v=1\\ v\neq i \end{array}}^{C}m_{uv}}{\sum_{\begin{array}{c} u=1\\ u\neq i \end{array}}^{C}e_{u}}$	$\frac{\sum_{i=1}^{C}NPV_{i}}{C}$
Accuracy ACC	Not defined	$\frac{\sum_{i=1}^{C}m_{ii}}{m}$
$F_{1}$	$2\frac{PRC_{i}\cdot SNS_{i}}{PRC_{i}+SNS_{i}}$	$\frac{\sum_{i=1}^{C}F_{1i}}{C}$
Geometric Mean GM	$\sqrt{SNS_{i}\cdot SPC_{i}}$	$\frac{\sum_{i=1}^{C}GM_{i}}{C}$
Matthews Correlation Coefficient MCC	Not defined	$\frac{\sum_{i=1}^{C}\sum_{j=1}^{C}\sum_{k=1}^{C}\left(m_{jj}\;m_{ki}-m_{ij}m_{ik}\right)}{\sqrt{\sum_{i=1}^{C}\left(\sum_{j=1}^{C}m_{ij}\right)\left(\sum_{\begin{array}{c} i=1\\ v\neq j \end{array}}^{C}m_{iv}\right)}\sqrt{\sum_{i=1}^{C}\left(\sum_{j=1}^{C}m_{ij}\right)\left(\sum_{\begin{array}{c} j=1\\ u\neq j \end{array}}^{C}m_{uj}\right)}}$
Bookmaker Informedness BM	$SNS_{i}+SPC_{i}-1$	$\frac{\sum_{i=1}^{C}BM_{i}}{C}$
Markedness MK	$PRC_{i}+NPV_{i}-1$	$\frac{\sum_{i=1}^{C}MK_{i}}{C}$

**Table 4:** Performance metrics for the procedure of counting frames.

	SNS	SPC	PRC	NPV	ACC	F1	GM	MCCn	BMn	MKn
MinDis	82.66%	92.37%	60.03%	90.51%	75.92%	62.89%	87.22%	82.08%	87.52%	75.27%
MaxLik	93.49%	97.72%	79.89%	96.79%	92.40%	84.88%	95.57%	93.84%	95.61%	88.34%
DecTr	82.51%	96.69%	95.30%	97.36%	92.40%	86.15%	88.04%	93.60%	89.60%	96.33%
kNN	89.64%	97.57%	95.97%	97.90%	94.35%	92.01%	93.11%	95.23%	93.60%	96.93%
LogReg	78.17%	92.67%	81.05%	93.49%	82.83%	76.74%	83.81%	86.01%	85.42%	87.27%
Neur	41.80%	77.90%	40.44%	77.24%	24.65%	22.46%	50.59%	55.86%	59.85%	58.84%
Discr	82.05%	93.70%	75.74%	93.80%	84.56%	76.14%	86.90%	87.17%	87.87%	84.77%
Bayes	79.32%	94.43%	81.15%	94.73%	86.52%	79.11%	85.32%	88.71%	86.87%	87.94%

**Table 5:** Confusion matrix using kNN frame classifier and the counting method.

		Classification Class			
		*Ep. cal.* Mating Call	*Ep. cal.* Release Call	*Al. ob.* Mating Call	*Al. ob.* Distress Call
Data class	Ep. cal. mating call	99.46%	0.54%	0%	0%
	Ep. cal. release call	30.16%	65.08%	4.77%	0%
	Al. ob. mating call	5.97%	0%	94.03%	0%
	Al. ob. distress call	0%	0%	0%	100%

**Table 6:** Performance metrics classifying the score series obtained by the kNN frame classifier.

	SNS	SPC	PRC	NPV	ACC	F1	GM	MCCn	BMn	MKn
Count	89.64%	97.57%	**95.97%**	97.90%	94.35%	92.01%	93.11%	95.23%	93.60%	96.93%
MinDis	95.27%	98.79%	95.01%	98.78%	96.89%	95.14%	96.97%	97.32%	97.03%	96.90%
MaxLik	91.54%	97.21%	82.23%	96.73%	92.17%	86.10%	94.32%	93.44%	94.37%	89.48%
DecTr	85.95%	97.40%	89.09%	97.58%	93.55%	87.42%	91.16%	94.41%	91.68%	93.33%
kNN	93.86%	98.48%	93.24%	98.53%	96.31%	93.53%	96.11%	96.82%	96.17%	95.89%
LogReg	93.39%	98.45%	91.24%	98.44%	96.20%	92.24%	95.85%	96.73%	95.92%	94.84%
Neur	79.72%	97.24%	83.59%	97.52%	92.97%	81.50%	87.43%	93.91%	88.48%	90.56%
Discr	94.48%	98.66%	94.29%	98.71%	96.66%	94.34%	96.47%	97.12%	96.57%	96.50%
Bayes	92.72%	97.62%	82.37%	97.10%	93.09%	86.59%	95.14%	94.24%	95.17%	89.73%

**Table 7:** Confusion matrix using the minimum-distance classifier operating on the score series obtained by the decision-tree frame classifier.

		Classification Class			
		*Ep. cal.* Mating Call	*Ep. cal.* Release Call	*Al. ob.* Mating Call	*Al. ob.* Distress Call
**Data class**	Ep. cal. mating call	96.48%	2.98%	0.27%	0.27%
	Ep. cal. release call	3.17%	95.24%	1.59%	0%
	Al. ob. mating call	1.19%	0.24%	98.33%	0.24%
	Al. ob. distress call	0%	0%	0%	100%

**Table 8:** Performance metrics for three pairs of (score, frame) classifiers.

	SNS	SPC	PRC	NPV	ACC	F1	GM	MCCn	BMn	MKn
Count-kNN	89.64%	97.57%	95.97%	97.90%	94.35%	92.01%	93.11%	95.23%	93.60%	96.93%
Count-DecTr	82.51%	96.69%	95.30%	97.36%	92.40%	86.15%	88.04%	93.60%	89.60%	96.33%
MinDis-DecTr	97.51%	99.12%	92.60%	98.88%	97.35%	94.88%	98.30%	97.75%	98.31%	95.74%

**Table 9:** Performance improvement (%) over the simpler counting methods.

Classifier	Statistic	SNS	SPC	PRC	NPV	ACC	F1	GM	MCCn	BMn	MKn
MinDis-DecTr vs. Count-kNN	Mean	5.98	1.26	−0.92	0.89	2.59	3.34	1.08	2.15	3.62	−0.02
	Conf. Int.	±3.56	±0.79	±3.06	±0.79	±1.96	±3.15	±2.4	±1.65	±2.06	±1.8
	Pr. Outperf.	99.9	99.9	28.2	99.1	99.9	98.6	99.9	99.9	99.9	48.2
MinDis-DecTr vs. Count-DecTr	Mean	12.98	2.15	−0.24	1.44	4.59	9.15	9.11	3.81	7.57	0.6
	Conf. Int.	±4.54	±0.84	±2.96	±0.79	±2.16	±4.04	±3.13	±1.7	±2.53	±1.83
	Pr. Outperf.	100	100	42.4	100	100	100	100	100	100	74.9
